# TLR4 as a negative regulator of keratinocyte proliferation

**DOI:** 10.1371/journal.pone.0185668

**Published:** 2017-10-05

**Authors:** Guergana Iotzova-Weiss, Sandra N. Freiberger, Pål Johansen, Jivko Kamarachev, Emmanuella Guenova, Piotr J. Dziunycz, Guillaume A. Roux, Johannes Neu, Günther F. L. Hofbauer

**Affiliations:** Department of Dermatology, University Hospital Zurich, Zurich, Switzerland; Universitatsklinikum Hamburg-Eppendorf, GERMANY

## Abstract

TLR4 is an innate immune receptor with expression in human skin, keratinocytes as well as squamous cell carcinoma (SCC) of the skin. In the present study we investigate the role of TLR4 as a negative regulator of keratinocyte proliferation. We present here that the expression of TLR4 increased with the differentiation of cultured keratinocytes in a passage-dependent manner or under calcium-rich conditions. Moreover, the down-regulation of TLR4 by specific knockdown increased the proliferation of HaCaT keratinocytes in vitro. In addition, subcutaneously injected HaCaT keratinocytes with shTLR4 formed growing tumors in nude mice. In contrast, we observed lower proliferation and increased migration in vitro of the SCC13 cell line stably overexpressing TLR4 in comparison to SCC13 TLR4 negative cells. In vivo, SCC13 TLR4-overexpressing tumors showed delayed growth in comparison to TLR4 negative tumors. The overexpression of TLR4 in SCC13 tumor cells was followed by phosphorylation of ERK1/2 and JNK and increased expression of ATF3. In gene expression arrays, the overexpression of TLR4 in tumor cells correlated with gene expression of ATF-3, IL-6, CDH13, CXCL-1 and TFPI. In summary, TLR4 negatively regulates the proliferation of keratinocytes and its overexpression reduces tumor growth of SCC cells.

## Introduction

The keratinocyte cell cycle is determined by proliferation and terminal differentiation, two processes that control and maintain the normal skin homeostasis. The basal skin layer is characterized by highly proliferative keratinocytes, expressing the differentiation markers K5 and K14. Well differentiated keratinocytes sustain the spinous, granular and corneal layers, have decreased proliferation potential and are characterized by the expression of K1, K10, involucrin, loricrin, filaggrin [1]; [2, 3]. The signaling cascades regulating the process of keratinocyte differentiation are dependent on the crosstalk between the different skin layers. Among the signaling pathways involved in keratinocyte differentiation are Notch [4]; Wnt [5] and p63, IRF6 [2, 6]. The dysregulation of these signaling pathways is observed in both inflammatory skin diseases such as psoriasis and non-melanoma skin cancer, such as SCC [7]; [8]; [9]; [10].

Toll like receptor 4 (TLR4) is a pattern recognition receptor and a key component of the innate immune system. TLR4 is also expressed in skin and cultured keratinocytes [11]; [12]. Of relevance for keratinocyte biology, it is reported that the adaptor protein TRIP (TRAF-interacting protein) regulates keratinocyte proliferation and differentiation [13]. The role of TLR4 is investigated in skin diseases such as dermatitis and psoriasis [14]; SCC [15] and melanoma [16], as well as in skin wound healing [17]; [18]. TLR4 signaling is reported to promote development of SCC in a MyD88-dependent manner and to be required for the recruitment of inflammatory cells during the carcinogenesis [19]. The development of epithelial tumors is also described through the interaction of TLR4 with HMGB-1 in the extracellular skin fluid causing the activation of NF-κB in keratinocytes. Thus, TLR4 mediates between inflammation and epithelial tumor development [20]. TLR4 is also reported to play a role in the prevention of chemically induced carcinogenesis through the activation of T cells [21]. The role of TLR4 in supporting cell growth or inducing apoptotic signals is presented mostly through the activation of other cell populations. However, a relation between TLR4 expression on keratinocytes and their proliferation has not been investigated by now. In the present study we propose a novel role for TLR4 as a regulator of keratinocyte proliferation.

## Materials and methods

The use of clinically indicated biopsy material for the study was approved by the Swiss ethics committee, Canton of Zürich, Switzerland. Participants provided verbal and written informed consent, approved by the Swiss ethics committee. Healthy and SCC skin was obtained from patients at plastic surgery following informed consent as approved by the ethical committee of the Canton of Zürich, Switzerland, and described previously [22]; [23]; [9]; [24].

### Immunohistochemistry (IHC)

TLR4 expression was analyzed in formalin-fixed paraffin-embedded skin samples obtained from the archives of the Dermatology Department of University Hospital Zurich (approval and agreement by the patients is documented). The tissue microarrays were composed of tumors from 63% male patients with a median age of 67 years. All tumors were from chronically sun-damaged skin, with the head the most frequent location in 56%. The expression was tested in normal skin (23 punch biopsies) and in moderately and well differentiated SCC skin derived from organ transplant recipients and immunocompetent patients (225 punch biopsies). The diagnosis and the classification of the SCC samples were performed by board-certified dermatopathologists by the criteria published by the WHO on tumors of the skin. The evaluation was performed by arbitrary evaluation of three board-certified dermatologists as well as one board-certified dermatopathologist, all co-authors of this manuscript. The staining intensity analysis was based on three selected areas chosen for the tissue microarray for every human tumor and on the whole tumor section for every single mouse tumor.

The staining was performed using anti-human TLR4 monoclonal mouse antibody (HTA 125, Abcam), anti human mouse ATF3 antibody (Santa Cruz Biotechnology) and anti IRF6 antibody (kindly provided by Prof. Paolo Dotto, Department of Biochemistry, University of Lausanne, Lausanne, Switzerland; Cutaneous Biology Research Center, Massachusetts General Hospital, Charlestown, MA, USA) following the manufacturers`protocol. All antibodies were used in a working dilution of 1:100. The negative and positive controls for the stainings were perfomed using sections from human placenta. The optimized staining conditions were used afterwards for the stainings of the normal skin sections as well of the TMA sections. The intensity of the staining was analyzed by arbitrary evaluation including the whole sample section and presented as arbitrary units respectively as weak, middle and strong staining The expression level (intensity) of TLR4, IRF6, ATF3 and the IRF6/TLR4 and ATF3/TLR4 correlation were evaluated using t-test and Anova. P-values below 0.0001 and 0.05 (p<0.05; p<0.0001) were considered as significant.

### Cell lines

The HaCaT cell line was obtained from Prof. Petra Boukamp, DKFZ, Heidelberg, Germany. The SCC13 cell line (epidermal malignant Squamous Cell Carcinoma cell line) is an immortalized cell line described by [25] and lacks expression of TLR4. For our experimental work it was kindly provided by Prof. Paolo Dotto, Department of Biochemistry, University of Lausanne, Lausanne, Switzerland; Cutaneous Biology Research Center, Massachusetts General Hospital, Charlestown, MA, USA.

Both HaCaT and SCC13 cell lines were grown in DMEM medium (Dulbeco`s Modified Eagle Medium; ThermoFisher Scientific) containing 10% FBS (ThermoFisher Scientific).

### Generation of primary keratinocyte cultures derived from healthy individuals and SCC patients

Primary normal and primary SCC-derived keratinocytes (SCC7) were generated in-house from normal skin (abdominoplastic reconstructive surgery) and biopsies from patients’ SCC samples. Single keratinocytes were isolated from 3–4 mm punch biopsies following the standard protocol for generation of primary keratinocyte cultures (CELLnTEC, Bern, Switzerland). For sufficient separation of the epidermis and dermis the punch biopsies were incubated overnight at 4°C in keratinocyte selection medium (Progenitor Cell Targeted (PCT) epidermal keratinocyte medium CnT07, low Ca2+, (CELLnTEC) with Dispase II (Roche). Afterwards the epidermal part of the skin was incubated in trypsin at room temperature for 30 min to insure efficient isolation of single keratinocytes. Freshly isolated cells were washed with medium and then transferred into CnT07 selection medium for continuous incubation (37°C, 5%CO2). The selection keratinocyte medium excludes a contamination with other skin cells (fibroblasts, melanocytes). All keratinocyte cultures were characterized as keratinocytes using the keratinocyte marker Keratin-14 (K14) as well as keratinocyte differentiation markers involucrin and filaggrin.

In contrast to the cell line SCC13, primary patient-derived SCC (SCC7) cells have a long but limited life span in culture.

### Immunoblotting

Differentiation of primary keratinocytes: Normal and SCC7 primary keratinocytes were grown in 6 cm dishes in 50% and full confluence (96 hours growth) or under high Ca^2+^ conditions (1.5mM CaCl_2_) in CnT07 keratinocyte medium (CELLnTEC, Bern, Switzerland). Cells were grown in the presence of Ca^2+^ for 24h, 48h and 96h. As positive control cells grown in a low Ca^2+^- medium for 96 hours was used where cells have reached full confluency and passage-driven differentiation. Cells were lysed in RIPA buffer (conventional protocol). The lysates were collected and subjected to SDS-PAGE, followed by immunoblotting using specific antibodies against human TLR4 (H-80, sc10741, 1:100 dilution), involucrin (sc-21748, 1:100 dilution), filaggrin (sc-66192, 1:100 dilution) and actin (sc-47778, 1:200 dilution), all from Santa Cruz Biotechnology. Incubation with primary antibodies was performed at 4°C, overnight. Incubation with secondary rabbit anti-mouse IgG H&L HRP (ab6728) was performed at room temperature for 1 hour. Proteins were detected by ECL on Hyperfilm (Amersham, GE Healthcare).

### LPS stimulation of SCC13 TLR4 overexpressing cells

SCC13 TLR4 overexpressing and SCC13 control cells were grown in 6 cm dishes in serum free DMEM medium for 24 hours. Afterwards cells were stimulated with ultrapure LPS (10μg/ml, Sigma) for different time intervals (0–60 min). Cells were lysed in RIPA buffer; the lysates were collected after each time interval and subjected to SDS-PAGE, followed by immunoblotting. The phosphorylated and non-phosphorylated form of ERK1/2 and JNK was detected by using specific rabbit polyclonal antibodies (anti-pp42/44 (#9101), anti-p42/44 (#9102); anti-rabbit HRP (#7074), all from Cell Signaling; anti-β-Actin (sc-47778, Santa Cruz Biotechnology, anti-mouse HRP (ab6728, Abcam). The expression of MyD88 (ab2064, Abcam) and IRAK-1 (D51G7, #4504, Cell signaling) upon LPS stimulation was analyzed using the same protein lysates. The secondary HRP-conjugated antibodies were used based on their species specificity to the primary antibodies.

ELISA: Cells were grown in 24 well plates till they reach 60% density. The expression of secreted IL-6 was analysed by ELISA (R&D Systems) before and after LPS stimulation at 6h and 24 hours. As controls unstimulated TLR4+ and TLR4- cells were used.

### Knockdown study

HaCaT keratinocytes were infected with lentiviral particles carrying specific shTLR4 consruct (sc- 40260-v) or shCtrl lentiviral particles (sc-108080), following the protocol conditions (Santa Cruz Biotechnology). Positive clones were selected by puromycine selection. The growth of the selected sh TLR4 and control clones, further grown under puromycine selection, was analyzed by BrdU proliferation assay.

### RNA extraction and qPCR

RNA was extracted using the TRIzol reagent (Invitrogen, Basel, Switzerland) following the protocol provided by the manufacturer. cDNA was synthesized from 1μg total RNA using a Reverse Transcription system kit (Promega). Specific primers for human TLR4 (Microsynth, Switzerland), (fwd 5‘ GCCCTGCGTGGAGGTGGTTC 3‘; rev 5‘ TGAGAAGGGGAGGTTGTCGGGG 3‘) were used. PCRs were performed by ViiA7 ^™^ Real-Time PCR System (Life Technologies) using FastStart Universal SYBR green Mix (ROX) (Roche).

### Overexpression study

SCC13 tumor cells were stably transfected with control (pUNO, Invivogen) and TLR4 expressing plasmid (pUNO-TLR4GFP, Invivogen) following the manufacturer’s protocol. For the selection of positive clones the transfected cells were further grown in DMEM medium containing blasticidine (10μg/ml) as a selection marker. The positive clones were visualized based on their GFP signal by fluorescent microscopy and FACS analysis. The cellular population with higher GFP signal, respectively higher TLR4 expression, were sorted using FACS sorter (FACSAria III) and further used in the study.

### Scratch assay

SCC13 TLR4+ and control cells were maintained under starving conditions (3% fetal calf serum) and treated with Mytomycine C (10 μg/ml) Sigma Aldrich) for 2 hours prior to the scratch assay. The scratch assay was performed on 100% confluent cells using a blue pipette tip for generating a cross region in every well. The number of migrated cells across the marked region was counted 15 hours after the scratch and presented as percent of control. The time point was chosen based on pilot experiments performed beforehand with several time-points where 15 hours was shown to discriminate most before cell death sets in.

### BrdU proliferation assay

Cells were seeded in 96 well plates in serum-free CnT07 or Dulbecco’s Modified Eagle Medium, DMEM in a cell density of 4x10^3^/well. Cell proliferation was measured using BrdU proliferation assay (Millipore) according to the manufacturer’s instruction. The analysis of cell proliferation was performed 24 hours after seeding. The percentage of cell proliferation was calculated using the equation (mean OD of treated cells/mean OD of control cells) X 100.

### Gene expression array

SCC13 TLR4 and SCC13 pUNO (control) cells were seeded in tetraplicates and treated with 10μg/ml ultrapure LPS for 24 hours. As controls untreated SCC13TLR4 and SCC13 pUNO cells were used. RNA was extracted by the TRIzol method and cDNA was generated by reverse transcription. The SurePrint G3 Human Gene Expression 8x60K (Agilent) with 50599 biological features was used to analyze the samples. Gene expression comparison was performed between untreated SCC13-TLR4 and control cells as well as between the LPS treated SCC13-TLR4 and control cells. Differentially expressed genes were selected to be relevant if the absolute log-fold change was more than 2 and significant when FDR adjusted p-value was less than 0.05 (NCBI, GEO Number: GSE89856). The gene expression was validated by PCR.

### Mice

The in vivo growth of HaCaT and SCC13 cell lines was analyzed in athymic nude mice (Hsd: Athymic Nude-Fox1^nu^/Foxn1^+^, Harlan).

#### Ethics statement

Mice were kept under specified pathogen-free conditions at the Institute of Laboratory Animal Sciences, University Hospital Zurich, and all procedures were approved by the ethical review committee of the cantonal veterinary office of Zurich (license ZH 104/2013).The animals were health checked the day after and four days after tumor cells injection. From second week onwards, the mice were controlled three days a week for health and tumor growth assessment. Animal well-being was monitored by observing behavior, activity, reactivity, breathing rate, fur appearance, and tumor growth/appearance. Body weight and tumor size were recorded weekly. Mice were euthanized with CO2, the air displacement being approx. 20–30% per minute. Criteria for termination used were: tumor diameter of 1.5 cm, necrotic or open lesions surrarounding the tumor, as well as weight reduction by more than 10%. Tumor excision was performed directly after euthanization and tumors were prepared for further in vitro analysis (PCR, immunochistochemistry, in vitro cell growth).

#### Growth of shTLR4 and csh HaCaT in nude mice

HaCaT cells stably transfected with shTLR4 or control sh were injected into nude mice subcutaneously (4x10^6^ cells/mouse; 6 mice per group). First measurement of the tumor size was performed 2 weeks after injection and approximately every 2 weeks further on. The tumor size was measured by caliper, and the tumor volume was calculated using (height X (width^2^))/100 formula, where width was the shorter distance. The differential growth between shTLR4 and control sh tumors was analyzed statistically for every time point and end day by t-test (p = 0.0002).

#### Growth of SCC13 TLR4 overexpressing cells in nude mice

SCC13 TLR4 overexpressing and control cells were injected subcutaneously in nude mice (4x10^6^ cells/mouse) in total of 22 animals divided into 3 groups. First measurement of the tumor size was performed 1 week after injection and approximately every week further on. The tumor size was measured by caliper, and the tumor volume was calculated using the (height X (width^2^))/100 formula, where width was the shorter distance. The animals were sacrificed, when the tumors reached either a volume of 1000mm^3^ or ulcerated. The differential growth between TLR4 overexpressing and control SCC13 tumors was analyzed based on the tumor volume for all tumors (p = 0.0192, Wilcoxon matched-pairs signed rank test). The smallest tumors measured were 100 mm^3^ at day 15 post injection (end day).

#### Immunochistochemical staining of the mice tumors

Hematoxylin staining and control staining for Ki67 (≠M7240,1:200 dilution), CD31 (#M0823, 1:40 dilution) and CD68 (#M0814,1:200 dilution) were performed using specific monoclonal mouse anti human antibodies (DAKO). Pre-treatment of the sections was performed using either Proteinase K (#S3020, DAKO) for CD31 and CD 68 stainings or boiling buffer for Ki67 staining (TRS 9,0, Epitope Retrieval Solution (10x Concentrate) ph 9,0, #RE 7119, Novocastra)

The staining procedure was performed by Real Detection Alkaline Phospatase system kit (#K005, DAKO) following the manufacturer`s protocol.

## Results

### Expression of TLR4, ATF3 and IRF6 in normal and SCC skin

**Fig 1:**
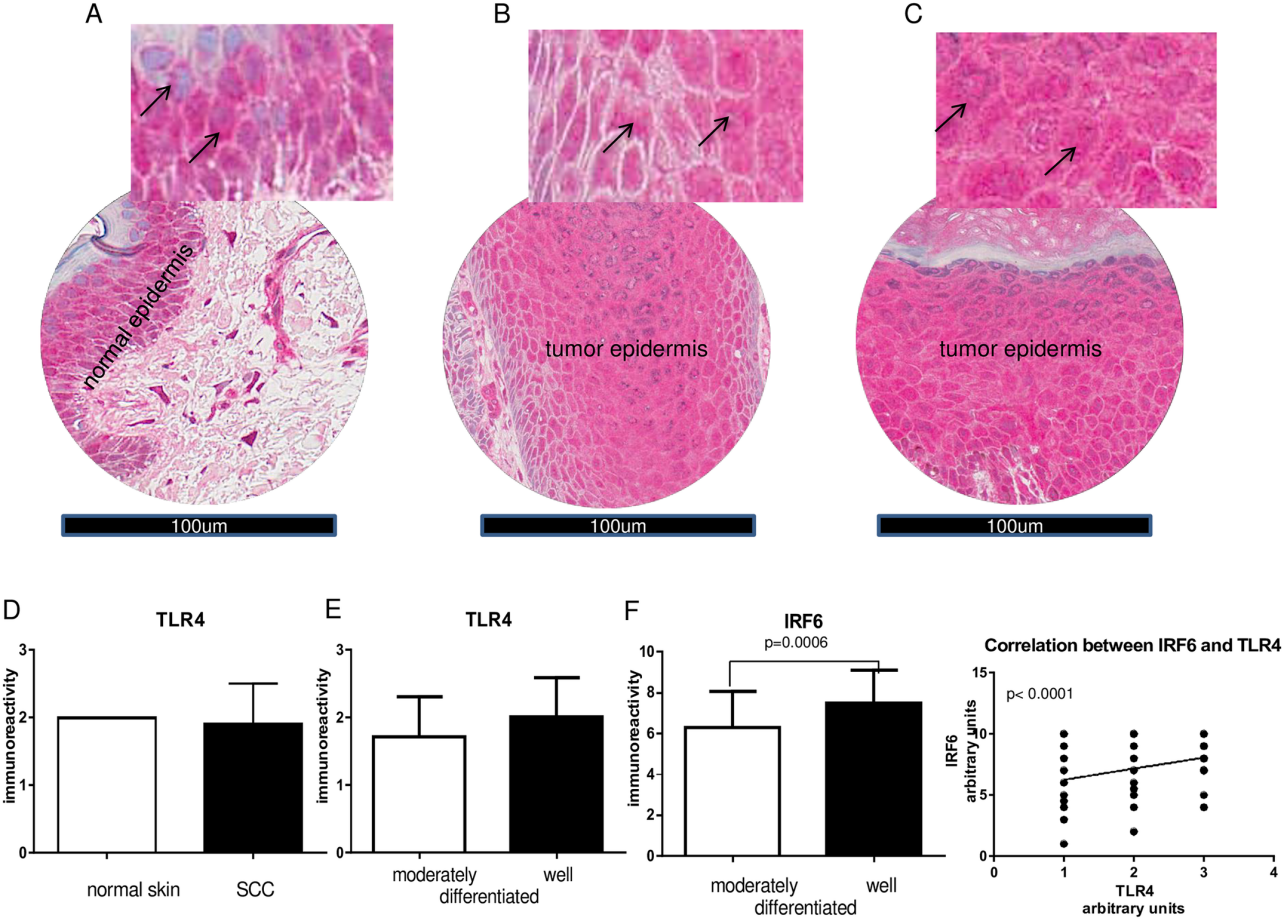
Expression of TLR4 in normal human and SCC skin. The expression level of TLR4 was analyzed on formalin-fixed paraffin-embedded skin samples and tissue microarray samples by immunochistochemistry using specific anti human TLR4 antibody (HTA125, dilution 1:100). The expression was tested in 225 SCC patient samples (moderately or well differentiated) and in 23 samples from normal skin. (A) Positive staining for TLR4 in normal epidermis. (B) Positive staining for TLR4 in moderately differentiated SCC. (C) Positive staining for TLR4 in well differentiated SCC. TLR4 positive keratinocytes show red intensity staining. TLR4 expression is visible in the membrane regions and perinuclear (cytoplasmic) regions and pointed by arrows. (D) Positive staining for TLR4 found in normal skin and maintained in SCC. (E) Positive staining for TLR4 is found in SCC in different stages of differentiation (moderately and well differentiated SCC). (F) Positive correlation between TLR4 and IRF6 expression (staining intensity) in moderately versus well differentiated SCC epidermis. The intensity of staining was analyzed by arbitrary evaluation and presented as arbitrary units respectively as weak, middle and strong staining. The expression level (intensity) of TLR4, IRF6 and the IRF6/TLR4 correlation were evaluated using t-test and Anova. P-values below 0.0001 and 0.05 (p<0.05; p<0.0001) were considered as significant.

The total TLR4 expression was analyzed by its immunoreactivity in the epidermis of normal and SCC-derived patient samples presented as tissue microarrays (TMA) and visualized by immunohistochemistry (Fig 1A–1C). TLR4 was expressed (red staining) in the perinuclear (cytoplasm and membrane) region with increasing staining intensity in the highly differentiated keratinocytes.

TLR4 expression was detected in both normal as well as moderately and well differentiated SCC epidermis (Fig 1D and 1E). The staining intensity of TLR4, IRF6 and ATF3 were arbitrarily rated as weak, middle and strong. We observed a correlation in the immunoreactivity between TLR4 and IRF6 and TLR4 and ATF3 in moderately versus well differentiated SCC samples (Fig 1F and 1D).

### TLR4 expression increases with the differentiation of normal keratinocytes in vitro

**Fig 2:**
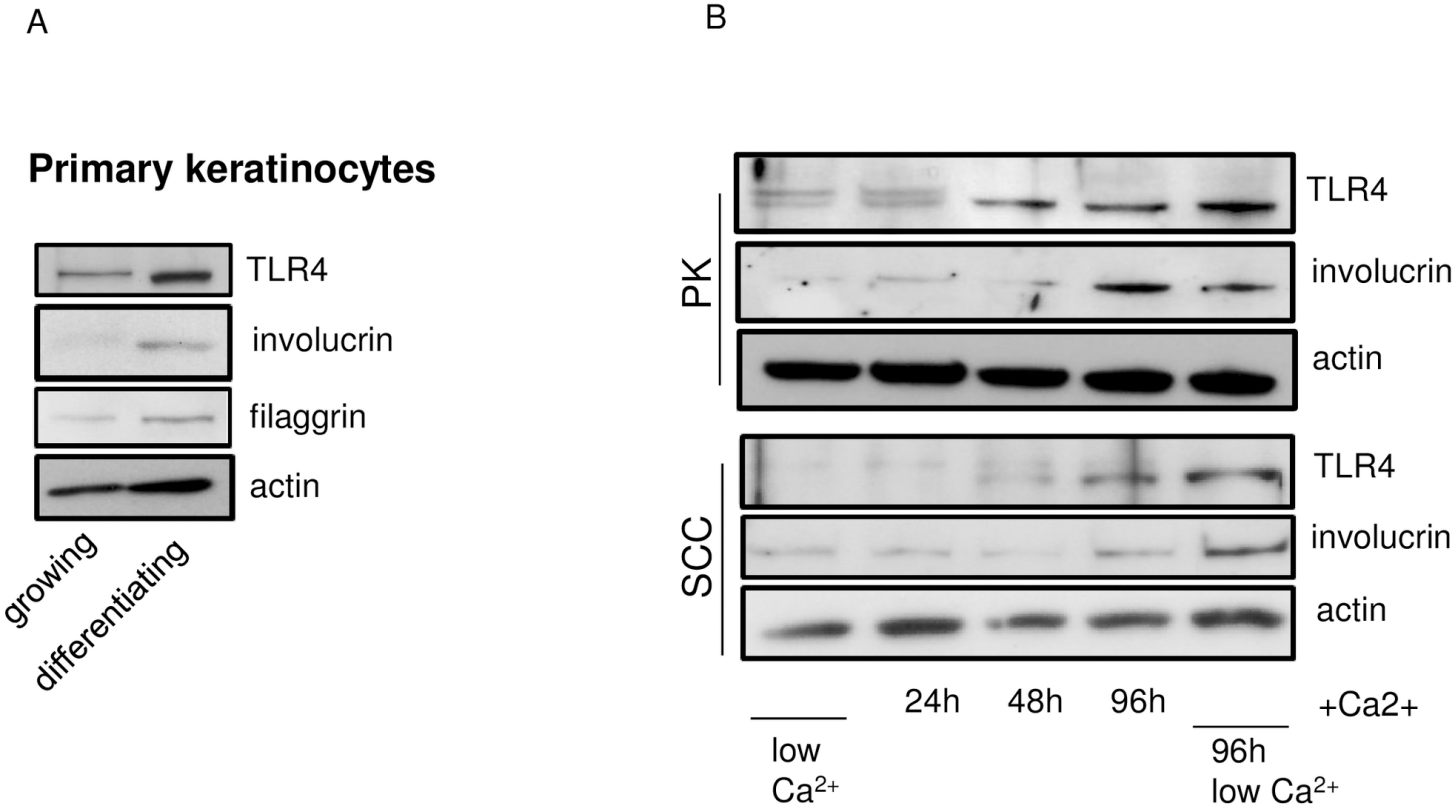
TLR4 expression increases with the differentiation of normal and SCC keratinocytes in vitro. (A) Primary keratinocytes were grown in duplicates (n = 2) till full confluency at 96h and the expression of TLR4 and keratinocyte differentiation markers (involucrin and filaggrin) was compared that of low passage (growing) keratinocytes by western blot using specific antibodies. The results were reproducible in two independent experiments. (B) Primary and SCC-derived keratinocytes were grown in duplicates (n = 2) in the presence of Ca^2+^ for different time intervals: 24h, 48h and 96 h. The group “no calcium” was used as positive control for differentiation at 96 hours, the time point of maximal differentiation induced by cell-cell contact. The expression of TLR4 and involucrin was analyzed by western blot using specific antibodies. The results were reproducible in two independent experiments.

The TLR4 expression was investigated in growing and differentiating normal primary keratinocytes. Differentiating keratinocytes were characterized by higher TLR4, involucrin and filaggrin expression (Fig 2A and S1 Fig). Similarly, increased TLR4 and involucrin expression was detected when primary and SCC-derived keratinocytes were driven to differentiate in Calcium-rich conditions in the time interval between 24h and 96h (Fig 2B and S2 Fig).

### Knockdown of TLR4 by specific lentiviral short-hairpin RNA (shRNA) induces keratinocyte proliferation

**Fig 3:**
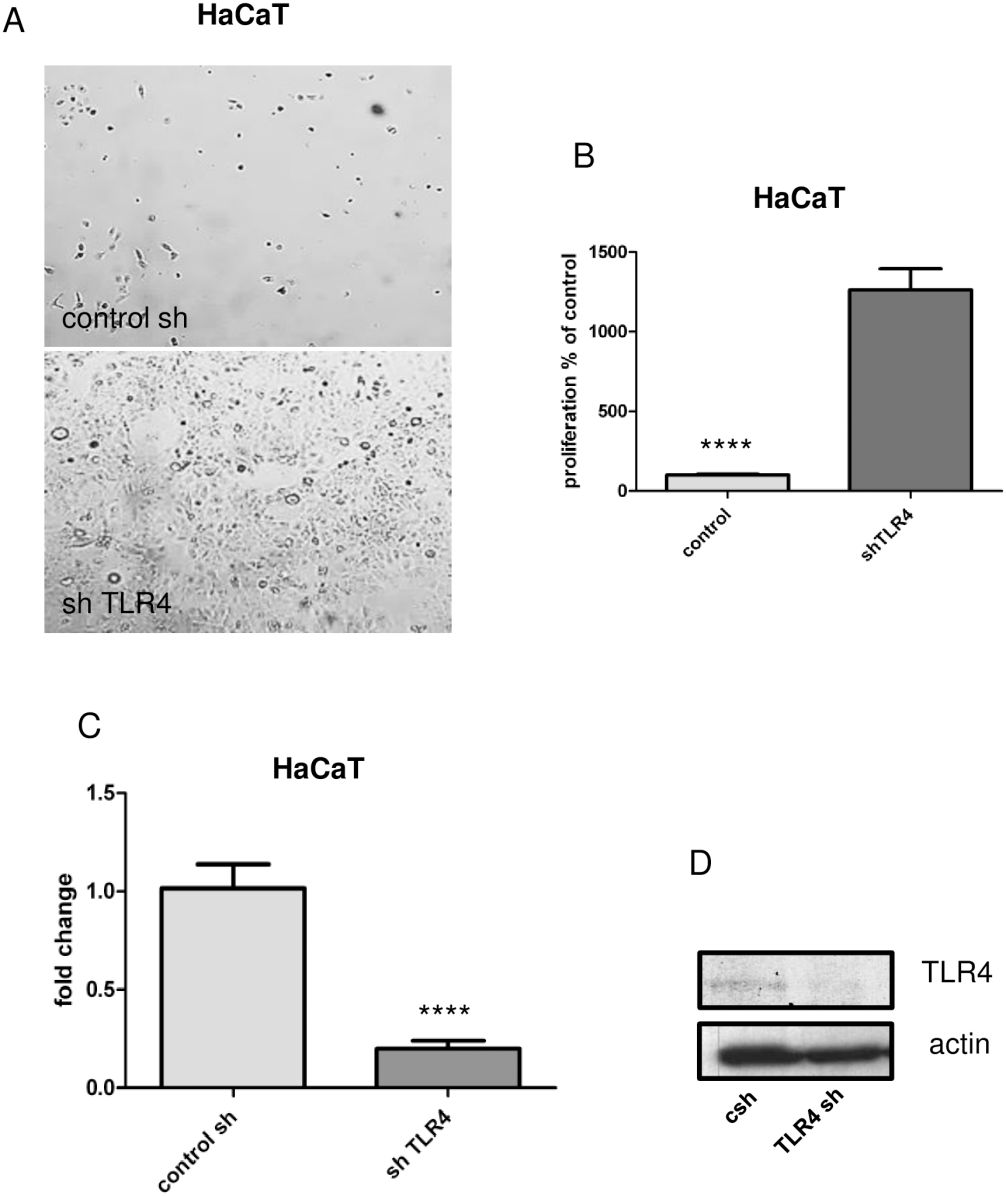
Knockdown of TLR4 induces proliferation of HaCaT keratinocytes. (A) Growth of sh control and shTLR4 cells under puromycine selection visualized by light microscopy. (B) Knockdown of TLR4 induces cellular proliferation. The alteration of the proliferation was analyzed by BrDU proliferation assay. The significance was evaluated by t-test analysis on duplicates (n = 2) from three independent experiments (p*<0.05; p****<0.0001). As a control cells transfected with a control sh were used. (C) Expression of TLR4 on transcriptional level after TLR4 knockdown. The differential TLR4 expression in the control and shTLR4 cells was evaluated by qPCR using specific TLR4 primers. (D) Expression of TLR4 on protein level after TLR4 knockdown. The differential TLR4 expression was evaluated by conventional western blot analysis using a monoclonal anti TLR4 antibody (HTA 125).

HaCaT keratinocytes were transfected in duplicates with a specific lentiviral shRNA against TLR4 and a control shRNA. Induction of keratinocyte proliferation after TLR4 knockdown was observed by BrDU proliferation assay (Fig 3A and 3B). The difference in cell proliferation between shTLR4 keratinocytes and control sh keratinocytes was evaluated by t-test on mean value from three independent experiments (p*<0.05; p****<0.0001), Fig 3B). The successful TLR4 knockdown in HaCaT keratinocytes was verified by PCR and immunoblotting (Fig 3C and 3D and S3 Fig).

### Growth of shTLR4 HaCaT keratinocytes in nude mice

**Fig 4:**
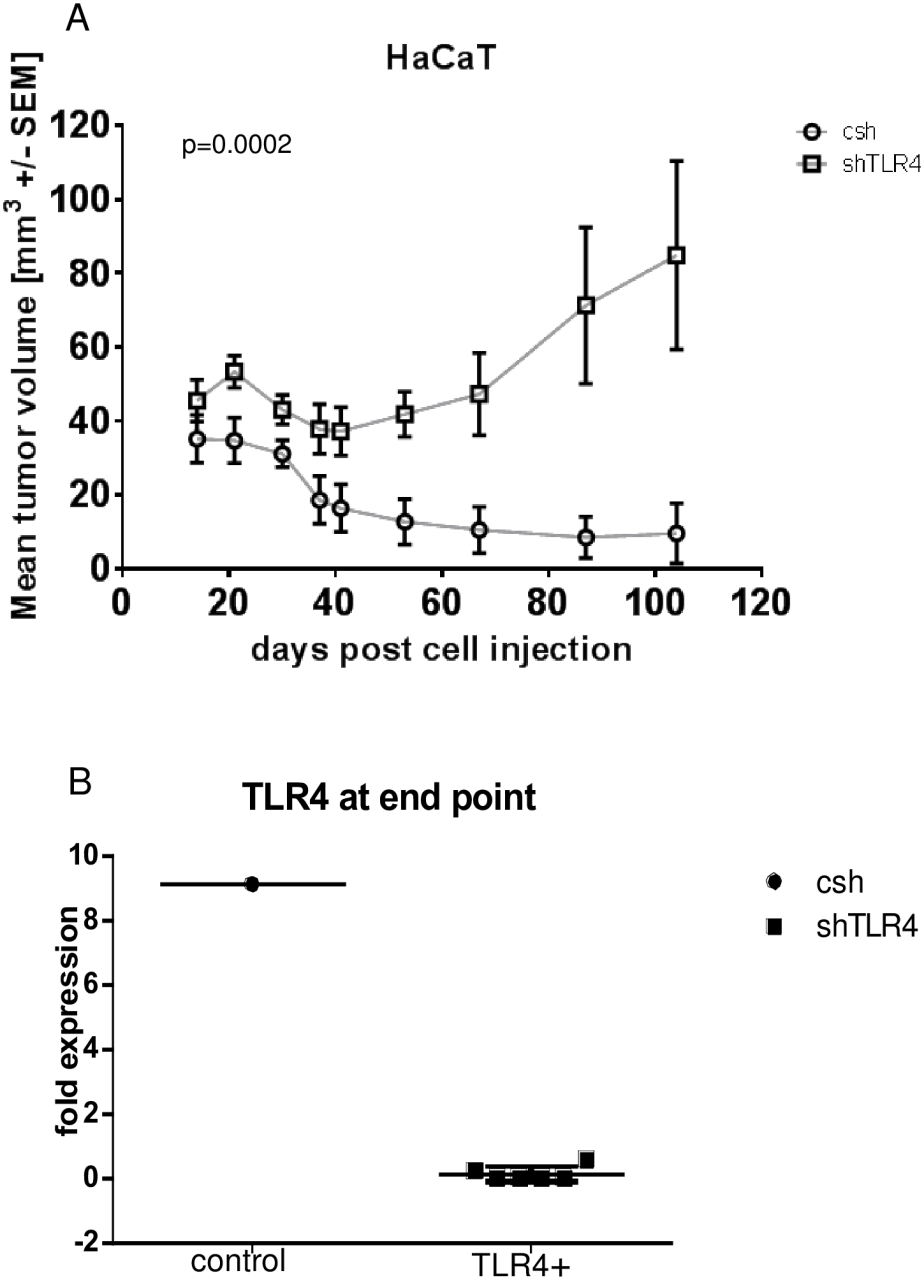
Growth of shTLR4 cells in nude mice. (A) Knockdown of TLR4 induces growth of HaCaT cells in nude mice (injected 4X10^3^ cells/mouse (mice, n = 6/group); p = 0.0002, t-test). The graphic represents the tumor growth (tumor volume) at different time intervals post injection. (B) Expression of TLR4 in the control and sh TLR4 tumors on end day. The differential TLR4 expression was evaluated on transcriptional level by qPCR using specific anti-human TLR4 primers. The tumor RNA and cDNA synthesis were performed using TRIzol reagent and RT respectively.

HaCaT shTLR4 keratinocytes were injected subcutaneously in nude mice and the growth of the tumors was investigated at different time points over 3 months. Mice injected with TLR4 knockdown cells developed progressively growing tumors in comparison to mice injected with control cells (difference at end point, p = 0.0002, t-test, Fig 4A). Only 1 out of 6 control tumors was still detectable 102 days after injection. This control tumor served as a basis for comparison of the TLR4 expression level (Fig 4B). Based on that any further histological or molecular biological comparison of the control and shTLR4 tumor tissue was not feasible.

### SCC13 TLR4 overexpressing cells show a delay in proliferation and higher migratory activity in vitro

**Fig 5:**
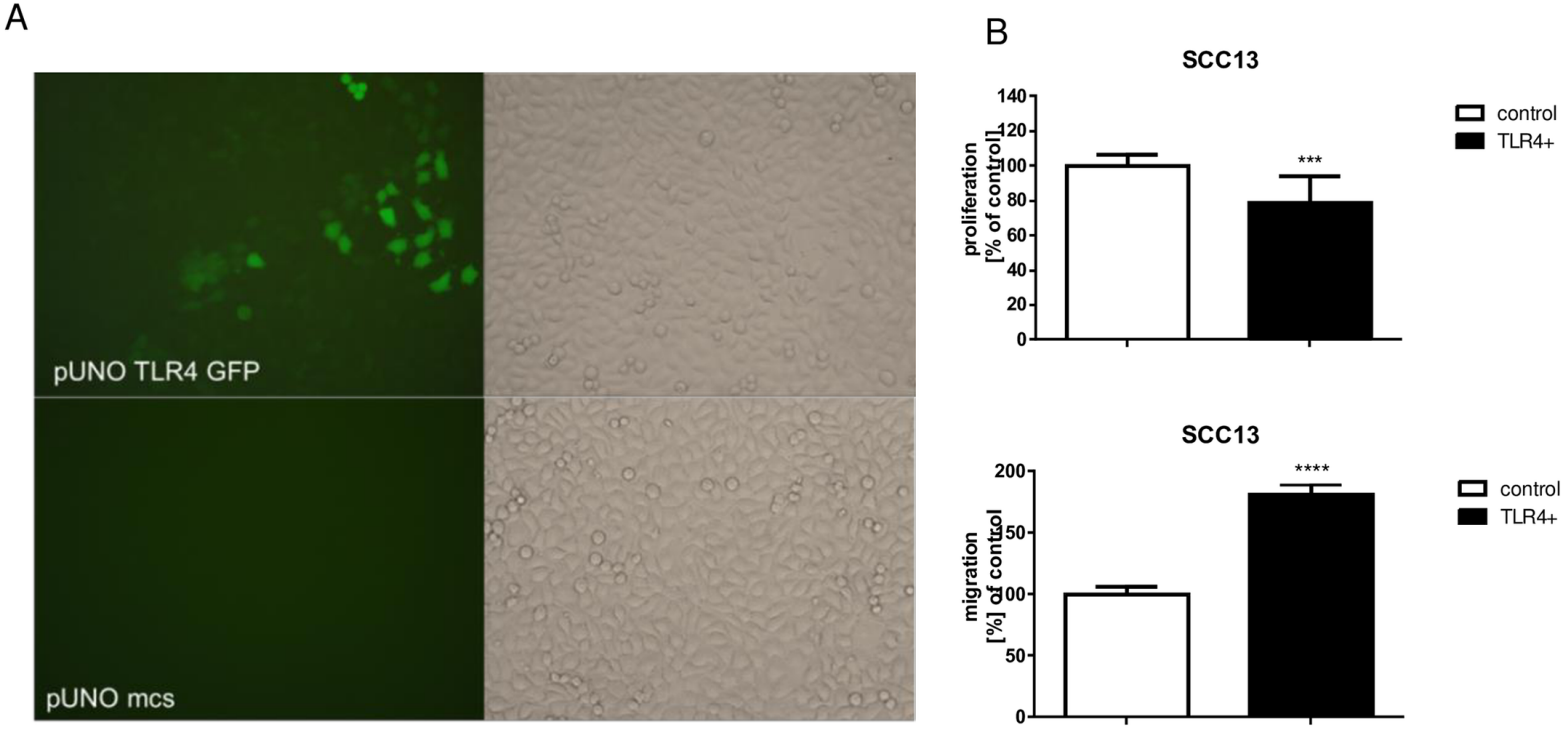
Overexpression of TLR4 in SCC13 tumor keratinocytes. (A) Visualization of SCC13-TLR4-GFP cells by fluorescent microscopy. SCC13 cells were stably transfected with pUNO-TLR4-GFP and control pUNO-mcs-expressing vectors. TLR4-GFP positive cells revealed nice green fluorescent staining in contrast to control cells. (B) SCC13-TLR4 cells show lower proliferation and higher migratory activity. The proliferation and migration of TLR overexpressing cells were investigated by BrDU proliferation assay and scratch assay and compared to cells expressing control vector only. The alterations in proliferation and migration were statistically significant (n = 4, two independent experiments, p*** = 0.0006, p****<0.0001, t-test).

SCC13 tumor cells were stably transfected with TLR4 expressing and control plasmid and maintained in culture under selection conditions (Blasticidine, 10μg/ml). The transfected TLR4-GFP cells showed differential intensity of the GFP signal. SCC13 with high TLR4-GFP signal were separated from the GFP-negative cells by FACS sorting. The sorted cells contained three populations of GFP positive cells and were distributed into three fractions. The third fraction (green peak) with highest GFP signal was used further in the study (Fig 5A and S4 Fig).

TLR4 overexpressing cells showed lower proliferation versus control as detected by BrdU proliferation assay (Fig 5B). Using conventional scratch assay (blocking proliferation by mitomycin C) we detected increased migration of TLR4 overexpressing SCC13 cells in comparison to control cells (Fig 5B).

### Growth of SCC13 TLR4 overexpressing cells in vivo

**Fig 6:**
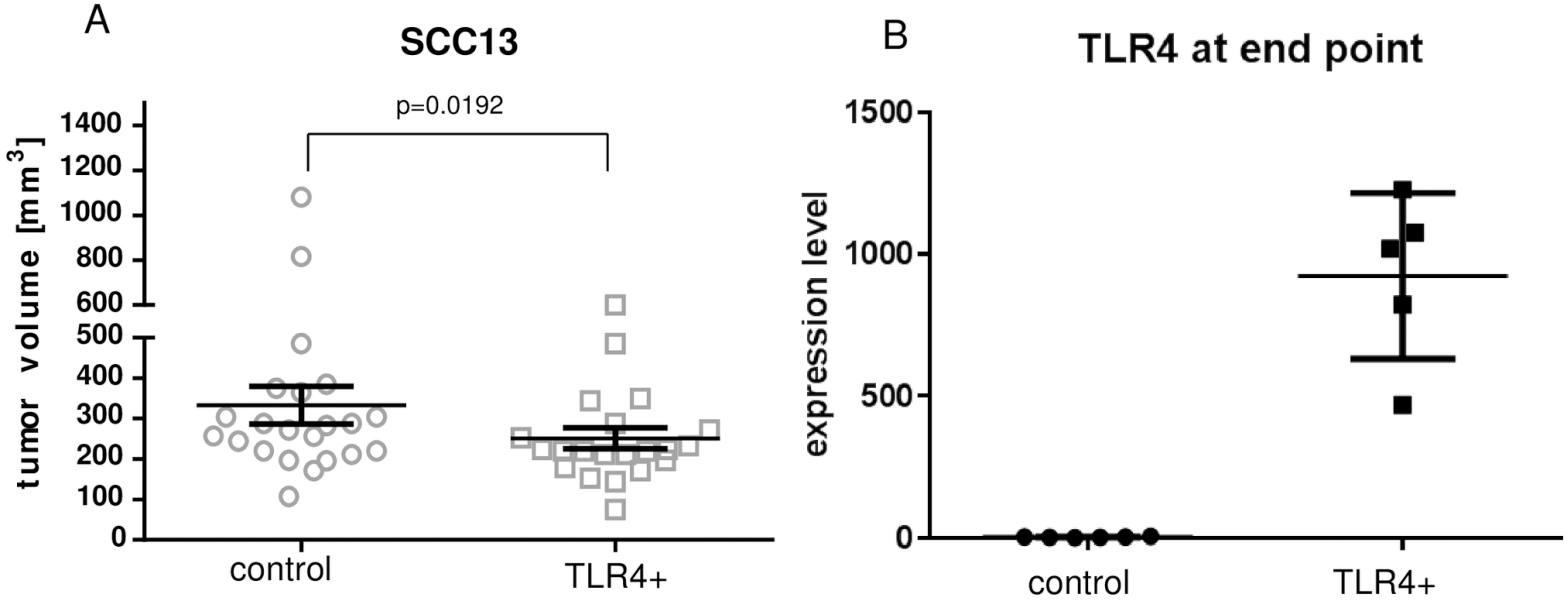
Delayed growth of SCC13-TLR4 overexpressing tumors. (A) Delayed growth of SCC13-TLR4 tumors in nude mice. The graph represents the tumor volume of the TLR4 and control tumors at day 15 after injection (end day, n = 22; p = 0.0192, Wilcoxon matched-pairs signed rank test). (B) Expression of TLR4 in SCC13-TLR4 and SCC13-pUNO control tumors at end day. The expression level of TLR4 in control and TLR4+ tumors was analyzed by qPCR using specific anti human TLR4 primers. The TLR4 overexpressing tumors (n = 22) retained high levels of TLR4 expression. The graph represents an example of TLR4 expression in animal group 1^st^ (n = 5).

SCC13-TLR4 and control cells were injected subcutaneously into nude mice. The tumor growth was analyzed based on tumor volume at different time points starting at 1 week after injection with following 10 and 15 days after injection (S5 Fig). SCC13-TLR4 tumors showed lower growth in comparison to control tumors: mean tumor volume: 350 mm^3^ (control) and 250 mm^3^ (TLR4 overexpressing tumors); p = 0.0192 Wilcoxon matched-pairs signed rank test, two-tailed, Fig 6A). The tumors were still overexpressing TLR4 at end day (15 days post injection) as detected by PCR (Fig 6B). The expression of keratinocyte differentiation markers was analyzed by qPCR. We detected a variable expression profile of involucrin, filaggrin and K1 within the tumors.

**Fig 7:**
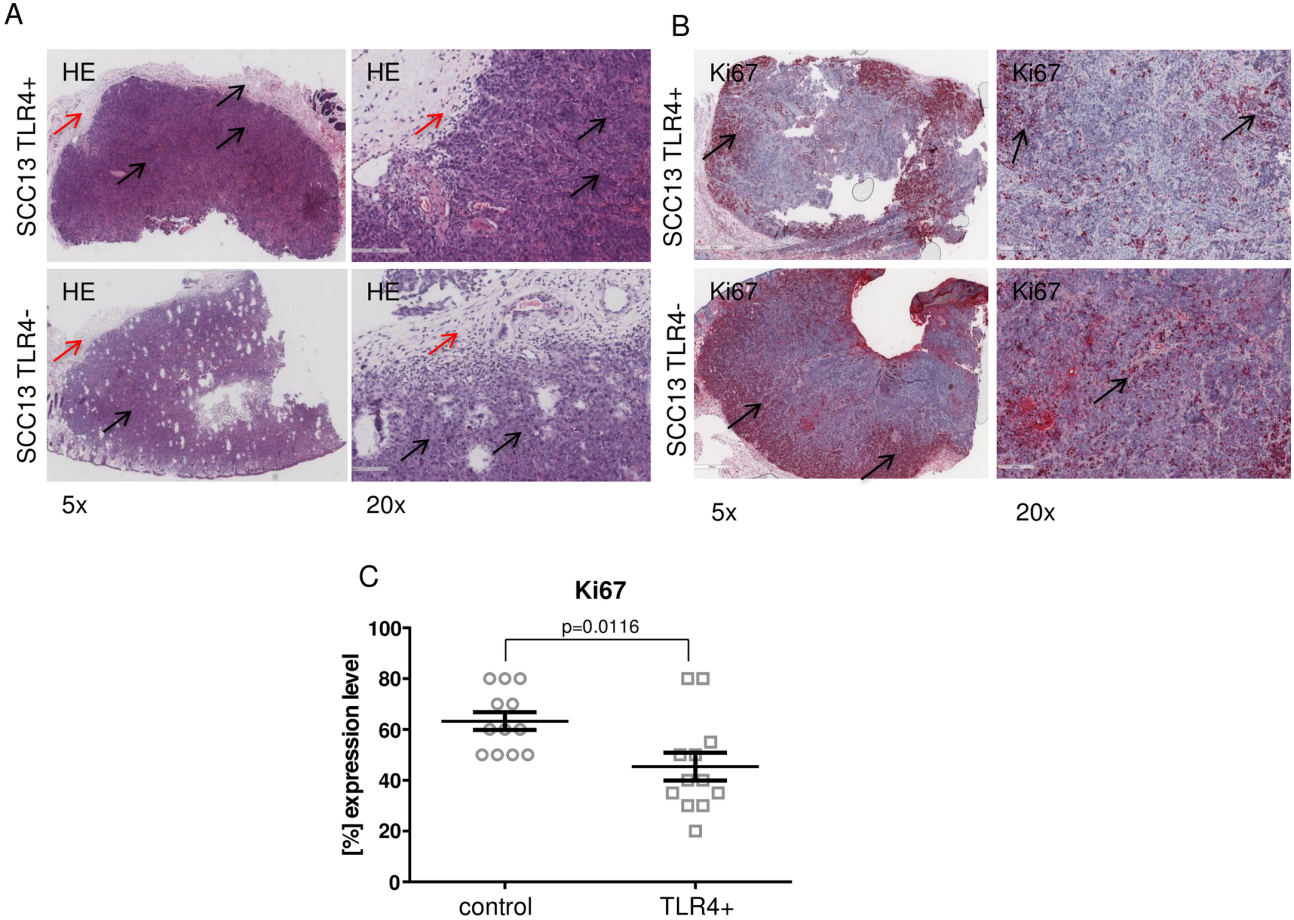
Immunochistochemical analysis of the SCC13 tumors. (A) Hematoxilyn staining (HE) for determining the tumor architecture. The tumor region and the tumor surrounding tissue (immune infiltrate) are marked with black and red arrows respectively. The staining was performed in all tumors and using the standard procedure for HE staining. (B) Staining for ki67 proliferation marker in all SCC13-TLR4 and control tumors. The cells expressing ki67 showed a positive red staining and are pointed with arrows. The intensity of the staining varies depending on the ki67 expression level. (C) Percentage of Ki67 positive cells in SCC13-TLR4 tumors versus control tumors. The differences in the expression level based on the immunochistochemical staining were quantified as [%] positive cell/area stained tissue (p = 0.0116, t-test, n = 12 sample group).

The tumor architecture of the excised tumors was analyzed by Hematoxilyn staining and showed a typical SCC tumor formation, with surrounding tumor infiltrate (Fig 7A). TLR4 overexpressing tumors showed also a lower expression of ki67 proliferation marker in comparison to control tumors (Fig 7B). Ki67 is a proliferation marker routinely used in pathology to measure the proliferative activity of cells (higher intensity staining correlating with higher proliferation) and little used in routine pathology for SCC. Rather, the measure of differentiation is a typical distinguishing feature and was thus chosen for grading of SCC in our tissue microarrays. In our mouse models, lacking the standard architecture of human tumors and thus without a proper measure for differentiation, we used Ki67 expression for detecting differential proliferation in SCC13-TLR4 overexpressing versus SCC13 control tumors.

The differences in the Ki67 expression level between TLR4 overexpressing tumors and control tumors were presented as [%] positive cells in the tumor area Ki67 positive cells were counted out of all cells in the whole section of TLR4 overexpressing and control tumors. Afterwards a percentage of the number of such positive cells based on the total number of cells was calculated (Fig 7C).

The staining intensity of Ki67 was evaluated using t-test. P-values below 0.05 (p<0.05) were considered as significant.

**Fig 8:**
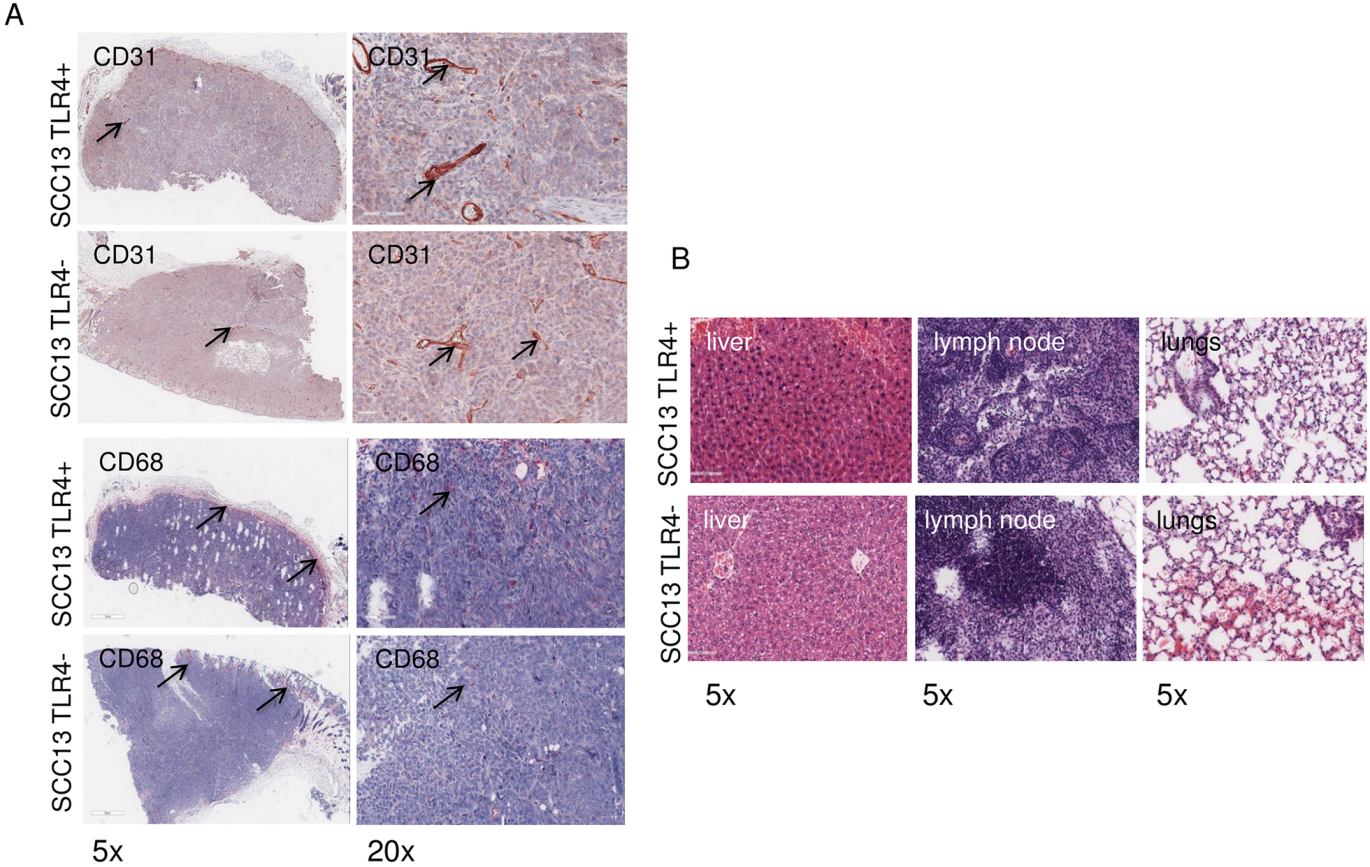
Immunochistochemical analysis of the SCC13 tumor vascularization, immune infiltration and metastasis. (A) Staining for CD31 and CD68 expression. The red positive signal corresponds to the expression of CD31 in the endothelial cells and the blood vessels respectively (black arrows). CD68 positive cells (macrophages) are stained in red and are present in the surrounding tumor infiltrate and the peripheral tumor area (black arrows). (B) Hematoxilyn staining for determining the organ-specific cellular architecture. The stainings were performed using specific antibodies against human Ki67, CD31 and CD68. All images are presented at low (5X) and highest (20X) magnification.

The tumor tissues were further investigated for detection of tumor vascularization, immune infiltration and metastasis in internal organs using immunohistochemistry. We did not detect any significant difference in the staining intensity of CD31 and CD68 in the tumor infiltrate between the TLR4 tumors and control tumors (Fig 8A). We did not detect also any tumor cells in the surrounding lymph nodes, lung or liver of the mice on end day (Fig 8B).

Extended time points for investigation of metastatic potential were not possible, as fast tumor growth combined with tumor ulceration required us to sacrifice animals as by the law.

### Functional relevance of TLR4 in SCC13 TLR4 expressing cells

#### Expression of pERK, pJNK and ATF3 in TLR4 overexpressing SCC13 cells

**Fig 9:**
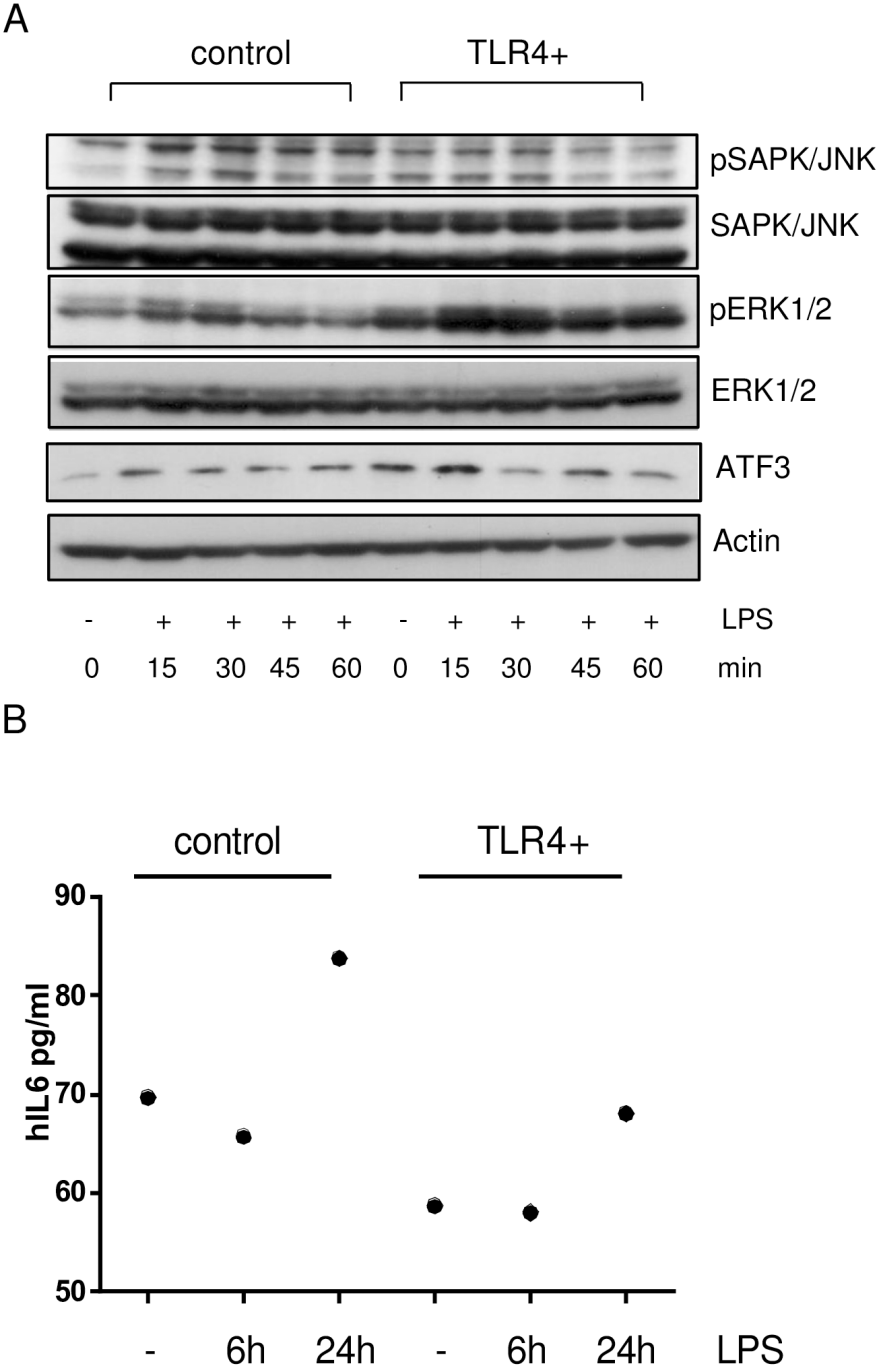
Functional relevance of TLR4 in SCC13-TLR4 cells. (A) Altered expression profile of pERK, pJNK and ATF3 in SCC13-TLR4 cells. The functionality of TLR4 was investigated upon LPS treatment for different time points and the differential expression/ phosphorylation profile was analyzed by western blot using specific antibodies. The phosphorylation of ERK and JNK was induced at 15–30 min after LPS treatment in both of the cell lines. SCC13-TLR4 cells showed increased phosphorylation of ERK and JNK in LPS-free conditions in comparison to SCC13-pUNO cells. SCC13-TLR4 cells show an increased ATF3 expression in LPS-free conditions in comparison to SCC13-pUNO control cells. The ATF3 expression was induced at 15 min after LPS stimulation in both of the cell lines. The results represent an example of two independent LPS treatments. (B) Alteration of IL-6 secretion in SCC13-TLR4 cells. SCC13-TLR4 and control SCC13-pUNO- cells were seeded in duplicates (n = 2) and induced by LPS at time points 15, 30, 45 and 60 min. Six and 24h after LPS stimulation cell supernatants were collected and analysed for IL-6 expression using ELISA and compared to untreated cells. LPS untreated SCC13-TLR4 cells show lower levels of IL6 in the cellular supernatant in comparison to control SCC13-TLR4 cells.

The functional relevance of TLR4 was tested by LPS stimulation. We analyzed the phosphorylation of ERK and JNK at different time points after LPS stimulation (Fig 9A and S6 Fig). We detected increased basal phosphorylation of JNK in unstimulated TLR4 overexpressing SCC13 cells in comparison to control cells. The increased phosphorylation was still detectable 15 min after LPS stimulation and decreased further in the course of LPS treatment. Similarly, increased phosphorylation of ERK in TLR4 overexpressing cells was observed in LPS-free conditions, which in contrast to JNK was inducible in presence of LPS. LPS also induced the expression of ATF3 in TLR4+ cells 15 minutes after treatment. SCC13 TLR4 cells showed lower, but LPS inducible IL-6 secretion in comparison to untreated cells (Fig 9B). We did not detect any alteration of pIRAK1 or MyD88 expression.

### Overexpression of TLR4 leads to differential gene expression in SCC13 cells

**Fig 10:**
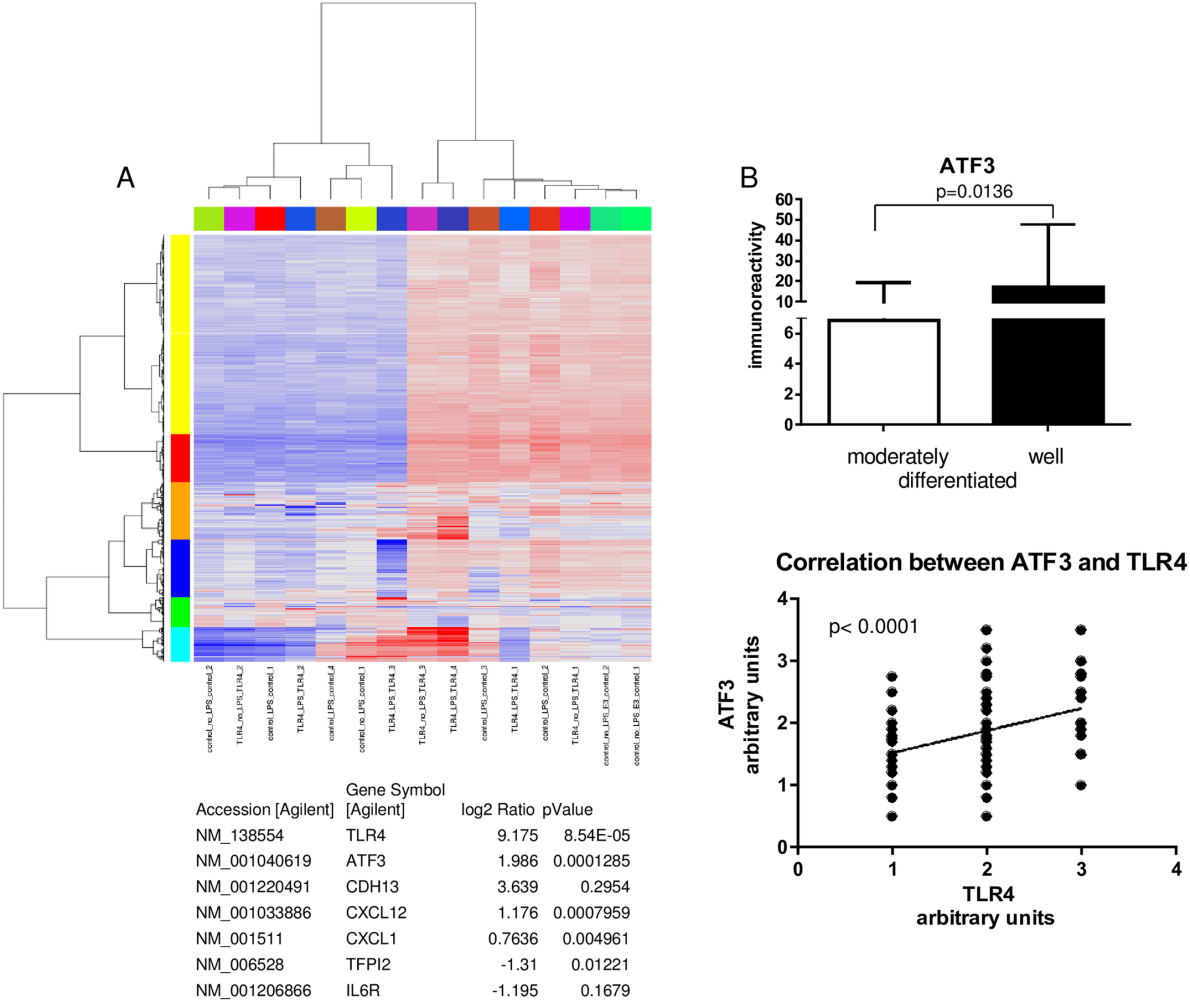
Altered gene expression in SCC13-TLR4 versus SCC13-pUNO control cells revealed by microarray analysis. Gene expression array: SCC13-TLR4 and control SCC13-pUNO- cells were seeded in tetraplicates (n = 4) and induced by LPS for 24 hours. The SurePrint G3 Human Gene Expression 8x60K (Agilent) with 50599 biological features was used to analyze the samples. Differentially expressed genes were selected as significant if the absolute log-fold change was more than 2 and FDR adjusted p-value was less than 0.05. (A) Clustering of significant probes and GO Analysis. Red GO categories are overrepresented among the significantly upregulated genes. Blue GO categories are overrepresented among the significantly downregulated genes. Black GO categories are overrepresented among all signifcantly regulated genes. The genes of interest with validated expression are presented in the table. (B) Positive correlation between TLR4 and ATF3 expression in moderately versus well differentiated SCC epidermis. The intensity of staining was analyzed by arbitrary evaluation and presented as arbitrary units respectively as weak, middle and strong staining. The expression level (intensity) of TLR4, ATF3, and the ATF3/TLR4 correlation were evaluated using t-test and Anova. P-values below 0.0001 and 0.05 (p<0.05; p<0.0001) were considered as significant.

The gene expression profile between SCC13-TLR4 and control SCC13-pUNO cells in presence or absence of LPS was investigated by cDNA microarray analysis. Upregulated and downregulated genes of interest were selected based on their statistically significant alteration in the expression level and according to their relevance in cell proliferation and migration in skin malignancy (Fig 10A). Within the upregulated genes were ATF3, CDH13 and CXCL-1,-12 and within the downregulated genes were IL-6, IL6R and TFPI. ATF3 upregulation in unstimulated SCC cells was confirmed on a protein level and its expression showed positive correlation to the TLR4 expression in patient SCC samples (Figs 9A and 10 and S7 Fig). The downregulation of IL-6R correlated with the downregulation of the secreted IL6 in LPS free conditions as revealed by ELISA (Fig 9B).

The gene expression profile of LPS treated SCC13-TLR4 cells did not reveal significant alterations in comparison to the LPS untreated cells.

## Discussion

In this study we investigate the role of TLR4 as a regulator of keratinocyte proliferation. We first analyzed the total expression of TLR4 in normal and SCC skin in TMA sections, where we detected immunoreactivity for TLR4 for all samples (Fig 1). The results shown in Fig 1E confirmed that TLR4 is expressed in both moderately and well differentiated SCC.

Interestingly, we observed a positive correlation between the expression of TLR4 and IRF6 in moderately versus well differentiated SCC keratinocytes (Fig 1F). This suggests that TLR4 expression increases with the differentiation of normal and SCC epidermis, as IRF6 is a transcription factor involved in epithelial differentiation [9, 26].

Such a correlation between the level of TLR4 and keratinocyte differentiation was also observed in an in vitro study, where the differentiation of normal and SCC keratinocytes was driven by known differentiation agents, such as Ca2+ (Fig 2B). A relation between passage growth and TLR4 expression has been previously observed, however in HaCaT cell line [27].

Other publications used other antibodies which we did not compare side to side in our experiments. We do show, however, as other authors found for several skin disease conditions that in our tumors TLR4 immunoreactivity increases towards the lumen, i.e. upper parts of the epidermis [28]. Our samples of normal epidermis stained are from sun-damaged areas of the skin, thus not easily comparable to the ones used by other authors, but apt in our study of human SCC where we want to capture the difference between benign, sun-damaged skin and malignant skin cancer, SCC.

A direct functional relation between TLR4 and keratinocyte proliferation and differentiation has not been investigated by now. The basis of our study is the direct relationship between TLR4 expression and keratinocyte growth in the absence of external stimuli and dependent on the TLR4 levels and intracellular mechanisms controlling the keratinocyte lifespan only. In relation to that we present that knockdown of TLR4 induces proliferation of keratinocytes in vitro and in vivo (Figs 3 and 4). Hyperproliferation of keratinocytes is observed mostly in inflammatory skin diseases as psoriasis [29]; [30]. TLR4 is expressed in psoriatic skin [31]; [14], however, a direct regulation of proliferation by TLR4 in the keratinocytes has not been discussed. Studies in TLR4 deficient mice and in vitro studies revealed involvement of TLR4 in skin wound healing [17]; [32]. In contrast to our results the authors describe activation of TLR4 signaling upon immune stimuli, which induces keratinocyte proliferation. A direct involvement, however, of TLR4 expressed on keratinocytes as a negative regulator of keratinocyte proliferation is not discussed.

Our study suggests that TLR4 is also a regulator of migration, proliferation and growth of SCC tumors. We show that TLR4 overexpression reduces proliferation and induces migration of SCC13 cells in vitro and delays growth of SCC13 tumors in vivo (Figs 5 and 6). In addition to that, our results revealed that the tumor delay of TLR4 overexpressing tumors correlated with the lower expression of ki67 proliferation marker (Fig 7). Different phenotypes of cancer cells either accentuating migration or proliferation have been described previously [33]. We believe that our manipulation of TLR4 in SCC13 had a greater effect on migration than proliferation. However, the effect on proliferation and migration cannot be compared directly as for the migration assay the cells were maintained under starving conditions and proliferation was blocked additionally by mitomycin C.

SCC13 cells are a suitable model for investigation of TLR4 dependent proliferation, however, differentiation of SCC13 cells based on TLR4 expression proved difficult to ascertain due to an insufficient Notch signaling pathway in these cells [34]; [35].

Generally, SCC13 are known also for their high metastatic potential [36]; [37]. We, however, did not detect any tumor cells in lymph nodes or other organs of the treated animals (Fig 8B), possibly due to early termination of the experiments based on ulceration of the primary tumor, demanding animals to be sacrificed. The nude mice used in this study are suitable model for the investigation of skin tumor development. However, these mice are largely immunodeficient as they lack functional T and B cells. Therefore, additional investigations concerning immune response against the tumor, for example, were not feasible. Our findings focused on keratinocyte behavior in relation to TLR4, not an immunological interplay with the microenvironment.

Human patient material and in vivo models in rodents have been studied for the involvement of TLR4 in tumor progression, although the focus of these projects was mainly on the involvement of the innate immune system [38]; [19]; [21]; [39, 40]; [16]. Studies in TLR4-deficient mice revealed that TLR4 has a supportive role in the development of chemically induced skin cancer through immune response [21]; [19]. While these mice are globally deficient for TLR4 with a big impact on innate immunity to be expected, tumor cell cultures originating directly from the DMBA-induced skin tumors in TLR4-deficient mice, however, show an increased proliferative capacity in comparison to tumors of control mice in the absence of immune cells. This supports our data concerning TLR4 expression and keratinocytes proliferation and tumor growth.

We observed specific phosphorylation changes of pERK and pJNK dependent on the TLR4 levels in tumor keratinocytes (Fig 9A).

The phosphorylation pattern of ERK and JNK after LPS stimulation in TLR4 overexpressing cells confirms the functionality of TLR4 in our model. Based on our results we suggest a relationship of ERK and JNK pathways and TLR4 overexpression, possibly contributing to the balance of proliferation and differentiation. This balance might be additionally maintained by the expression of IL6. Interestingly, SCC13-TLR4 cells showed lower IL6 expression than control cells, but still inducible by LPS (Fig 9B). These results point to both TLR4–related IL6 expression and confirmation of TLR4 functionality in our model. IL6 is known to be involved in the metastatic and invasion properties of melanoma, SCC of the skin and Head and Neck SCC [41]; [42].

Furthermore, using gene expression analysis in SCC13-TLR4 cells we identified and validated genes reported to be involved in proliferation and migration of malignant cells (Fig 10A). Interestingly, among the upregulated genes was ATF3, which expression correlated with the TLR4 expression in SCC13 TLR4 cells (Fig 9A) and in the SCC TMA (Fig 10B). ATF3 is reported as transcription factor involved in the pathogenesis of epithelial cancer and psoriasis [36]; [23]; [24, 43]. In relation to that, ATF3 is known to be a negative regulator of IL6 [44] [45]; [46]. This correlates with our results showing that TLR4 overexpressing cells have increased ATF3 expression and decreased IL-6 secretion. Based on that we suggest that ATF3 together with TLR4 and IL6 might be involved as a negative regulator of keratinocyte proliferation. In contrast to our results, some reports show a protective role of ATF3 in SCC, however in their relation to immunosuppressive agents [23]; [24]

Another upregulated gene was CDH13 (Cadherin H), known to participate as a modulator of proliferation and migration in melanoma, SCC and BCC [47]; [48, 49] as well as CXCL proteins, involved in the control of epithelial-mesenchymal interaction in normal or malignant epithelial cells [50]. Together with IL-6, among the downregulated genes was TFPI. TFPI is known to be involved in the formation of matrix-rich vascular-like networks in melanoma [51, 52]; [53] and is used as a diagnostic marker for SCC [54].

Since these individual genes have all been described in cancer models and tissue, we reason their dependence on TLR4 expression as shown in our study supportive for the suggested role of TLR4 in tumor growth.

Altogether we assume that TLR4 impacts keratinocyte biology as a regulator of proliferation in normal and tumor keratinocytes and also in the migration of tumor cells. Thus TLR4 as negative regulator of keratinocyte proliferation may associate with the progression of SCC of the skin. A better understanding of the regulatory role for TLR4 will be the basis for a later use in a therapeutic setting to impair keratinocyte proliferation such as in squamous cell carcinoma of the skin and to induce keratinocyte proliferation such as in wound healing.

## Supporting information

S1 Fig Quantitative estimation of the expression of TLR4, Involucrin and filaggrin in normal primary keratinocytes. The quantitative estimation was based on calculating the spot density of the bands for TLR4, involucrin and filaggrin and compared to the spot intensity of the actin bands. This was performed by LI-COR ODYSSEY^®^ Fc Dual-Mode Imaging System and Image Studio Lite Program. All quantitative estimations represent the spot density on the entire western blot, which is representative for two independent experiments with reproducible result. (A) Quantitative estimation of the TLR4, involucrin and filaggrin expression in growing versus confluent primary normal keratinocytes (PK). The expression of TLR4, involucrin and filaggrin is presented as a ratio between the spot density (arbitrary units) of the bands for these proteins and the spot density of the corresponding actin bands. The differential expression of those proteins in confluent keratinocytes was compared to their expression level in growing cells and presented as “% of control” (“control” = growing cells). (B) Quantitative estimation of TLR4 and involucrin expression in primary normal keratinocytes (PK) before and after treatment with Ca^2+^. TLR4 expression was analyzed in low Ca conditions (0h and 96h) and at 24h, 48h and 96 hours after Ca^2+^ treatment (1.5mM CaCl_2_). The quantified expression of TLR4 and involucrin is presented as a ratio between the spot density (arbitrary units) of the TLR4 and involucrin bands and the spot density of the corresponding actin bands. The differential expression of TLR4 for every time point is compared to its expression level in untreated cells and presented as “% of control”.
(TIFF)

S2 Fig Quantitative estimation of TLR4 and involucrin expression in primary SCC keratinocytes before and after treatment with Ca^2+^. TLR4 expression was analyzed in low Ca conditions (0h and 96h) and at 24h, 48h and 96 hours after Ca^2+^ treatment (1.5mM CaCl_2_). The quantified expression of TLR4 and involucrin is presented as a ratio between the spot density (arbitrary units) of the TLR4 and involucrin bands and the spot density of the corresponding actin bands. The differential expression of TLR4 for every time point is compared to its expression level in untreated cells and presented as “% of control”.
(TIFF)

S3 Fig Densitometrical quantification of TLR4 level in shTLR4 and control protein samples. The quantitative estimation of the differential expression was based on calculating the spot density of the bands corresponding to sh control and shTLR4 bands. This was performed by *Image J* software programm.
(TIFF)

S4 Fig SCC13 cells express TLR4-GFP. SCC13 stably transfected with TLR4-GFP show different populations of cells according to their GFP signal. SCC13 with high TLR4-GFP signal were separated from the negative TLR4-GFP population by FACS sorting, using FACSAria III. The FACS diagrams show the cellular distribution according to the GFP signal before and after sorting. The sorted cells contained three populations of cells according to their GFP signal and were distributed into three fractions. The third fraction (green peak) with highest GFP signal was used further in the study.
(TIFF)

S5 Fig Delayed growth of SCC13-TLR4 overexpressing tumors in comparison to control tumors during the time course. SCC13 TLR4 overexpressing and control cells were injected subcutaneously in nude mice (4x10^6^ cells/mouse). Tumor volume was measured preliminary at 1 week, 10 days and 15 days after injection. The graph represents preliminary tumor growth in sample groups 1^st^ and 2^nd^ pooled together, n-13).
(TIFF)

S6 Fig Quantitative estimation of of pERK and pJNK before and after LPS treatment. SCC13 TLR4 overexpressing and SCC13pUNO control cells were treated with 10μg/ml ultrapure LPS in a time course of 15min, 35min, 45 min and 60 min. The expression of pERK /ERK and pJNK/JNK was analyzed by western blotting. All quantitative estimations represent the spot density on the entire western blot, which is representative for two independent experiments with reproducible result. (A) Quantitative estimation of pERK before and after LPS treatment. The quantified expression of pERK and ERK is presented as a ratio between the spot density (arbitrary units) of pERK bands and the spot density of the corresponding ERK bands. The differential expression of pERK for every time point is compared to its expression level in untreated cells and presented as “% of control”. (B) Quantitative estimation of pJNK before and after LPS treatment. The quantified expression of pJNK and JNK is presented as a ratio between the spot density (arbitrary units) of pJNK bands and the spot density of the corresponding JNK bands. The differential expression of pJNK for every time point is compared to its expression level in untreated cells and presented as “% of control”.
(TIFF)

S7 Fig Quantitative estimation of ATF3 before and after LPS treatment. The quantified expression of ATF3 is presented as a ratio between the spot density (arbitrary units) of the ATF3 bands and the spot density of the corresponding actin bands. The differential expression of ATF3 for every time point is compared to its expression level in untreated cells and presented as “% of control”.
(TIFF)

S8 Fig Control immunochistochemical staining. (A) Positive control staining for TLR4 expression in human placenta. The TLR4 detection antibody (HTA 125) was used in a dilution 1:100. The images are presented at magnifications 5X and 20X. The positive red staining represents the TLR4 expression. (B) Negative control staining. The negative control staining was performed on human placenta and in the absence of the TLR4 antibody. (C) Differential TLR4 expression in normal epidermis. The basal keratinocyte layer is characterized by weak red intensity staining for TLR4 (red arrows). Upper and high-differentiated layers show stronger red intensity staining for TLR4 (black arrows).
(TIFF)

## Abbreviations

ATF3: Cyclin-AMP-dependent transcription factor 3
CDH13: H cadherin 13
CXCL-1: Chemokine (C-X-C motif) ligand 1
ERK: Extracellular-signal Regulated Kinases
JNK: cJUN-N-terminal kinase
MyD88: Myeloid differentiation primary response 88
SCC: Squamous Cell Carcinoma
TFPI: Tissue factor pathway inhibitor
TLR4: Toll-like receptor 4
