# Supplementary figures and images for: TLR4 as a negative regulator of keratinocyte proliferation
Source: PLoS One. 2017 Oct 5;12(10):e0185668. doi: 10.1371/journal.pone.0185668 (PMC5628845; doi:10.1371/journal.pone.0185668)

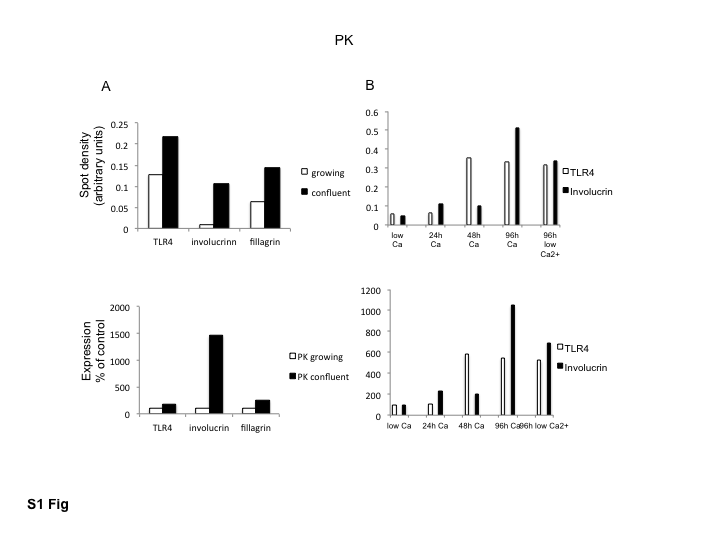

Supplement: S1 Fig — The quantitative estimation was based on calculating the spot density of the bands for TLR4, involucrin and filaggrin and compared to the spot intensity of the actin bands. This was performed by LI-COR ODYSSEY® Fc Dual-Mode Imaging System and Image Studio Lite Program. All quantitative estimations represent the spot density on the entire western blot, which is representative for two independent experiments with reproducible result. (A) Quantitative estimation of the TLR4, involucrin and filaggrin expression in growing versus confluent primary normal keratinocytes (PK). The expression of TLR4, involucrin and filaggrin is presented as a ratio between the spot density (arbitrary units) of the bands for these proteins and the spot density of the corresponding actin bands. The differential expression of those proteins in confluent keratinocytes was compared to their expression level in growing cells and presented as “% of control” (“control” = growing cells). (B) Quantitative estimation of TLR4 and involucrin expression in primary normal keratinocytes (PK) before and after treatment with Ca2+. TLR4 expression was analyzed in low Ca conditions (0h and 96h) and at 24h, 48h and 96 hours after Ca2+ treatment (1.5mM CaCl2). The quantified expression of TLR4 and involucrin is presented as a ratio between the spot density (arbitrary units) of the TLR4 and involucrin bands and the spot density of the corresponding actin bands. The differential expression of TLR4 for every time point is compared to its expression level in untreated cells and presented as “% of control”. (TIFF) [file pone.0185668.s001.tiff]

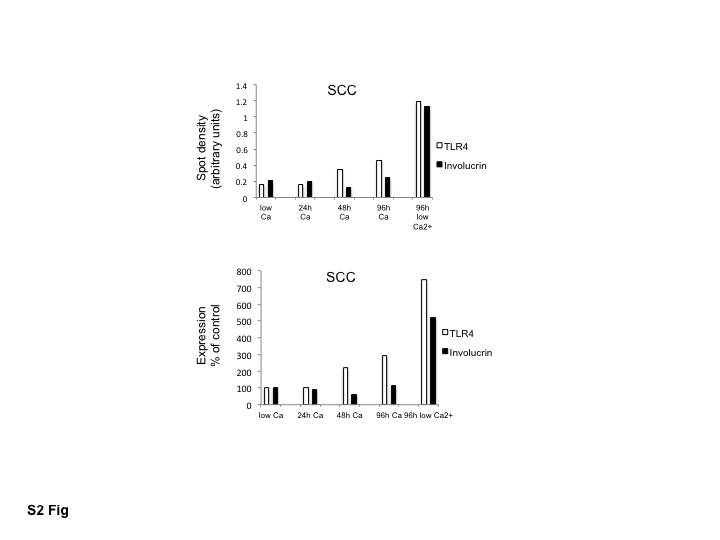

Supplement: S2 Fig — TLR4 expression was analyzed in low Ca conditions (0h and 96h) and at 24h, 48h and 96 hours after Ca2+ treatment (1.5mM CaCl2). The quantified expression of TLR4 and involucrin is presented as a ratio between the spot density (arbitrary units) of the TLR4 and involucrin bands and the spot density of the corresponding actin bands. The differential expression of TLR4 for every time point is compared to its expression level in untreated cells and presented as “% of control”. (TIFF) [file pone.0185668.s002.tiff]

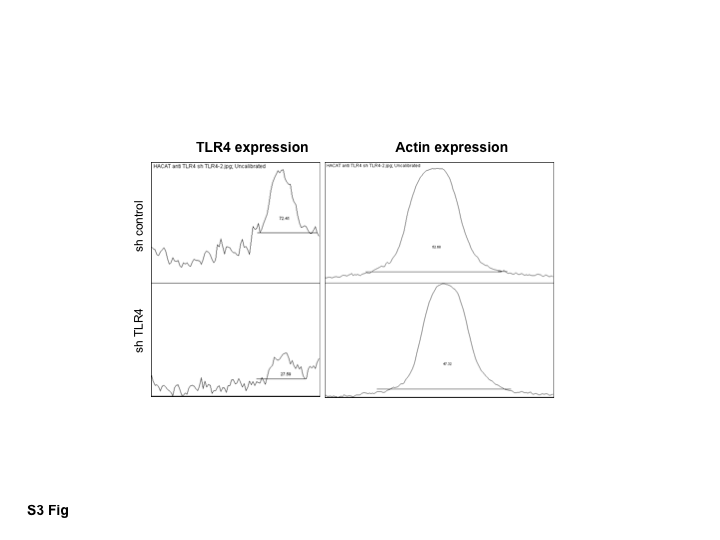

Supplement: S3 Fig — The quantitative estimation of the differential expression was based on calculating the spot density of the bands corresponding to sh control and shTLR4 bands. This was performed by Image J software programm. (TIFF) [file pone.0185668.s003.tiff]

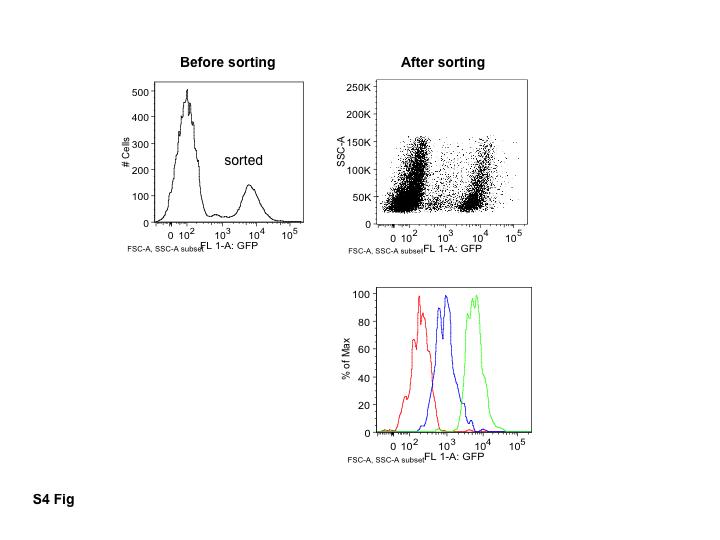

Supplement: S4 Fig — SCC13 stably transfected with TLR4-GFP show different populations of cells according to their GFP signal. SCC13 with high TLR4-GFP signal were separated from the negative TLR4-GFP population by FACS sorting, using FACSAria III. The FACS diagrams show the cellular distribution according to the GFP signal before and after sorting. The sorted cells contained three populations of cells according to their GFP signal and were distributed into three fractions. The third fraction (green peak) with highest GFP signal was used further in the study. (TIFF) [file pone.0185668.s004.tiff]

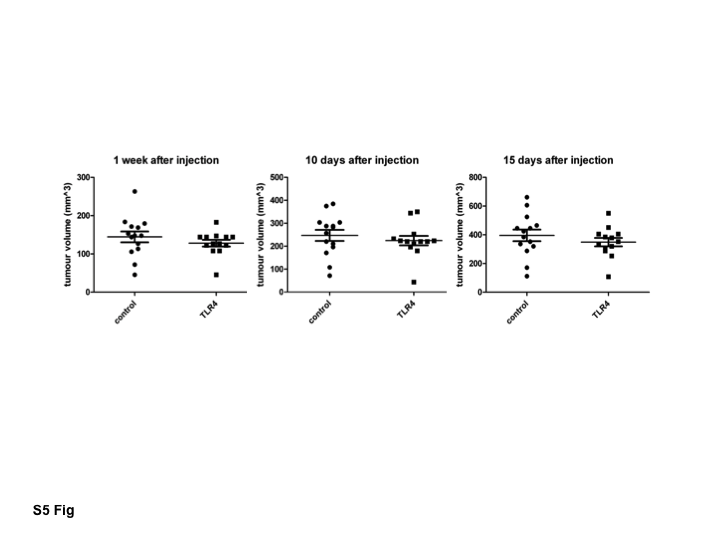

Supplement: S5 Fig — SCC13 TLR4 overexpressing and control cells were injected subcutaneously in nude mice (4x10^6^ cells/mouse). Tumor volume was measured preliminary at 1 week, 10 days and 15 days after injection. The graph represents preliminary tumor growth in sample groups 1st and 2nd pooled together, n-13). (TIFF) [file pone.0185668.s005.tiff]

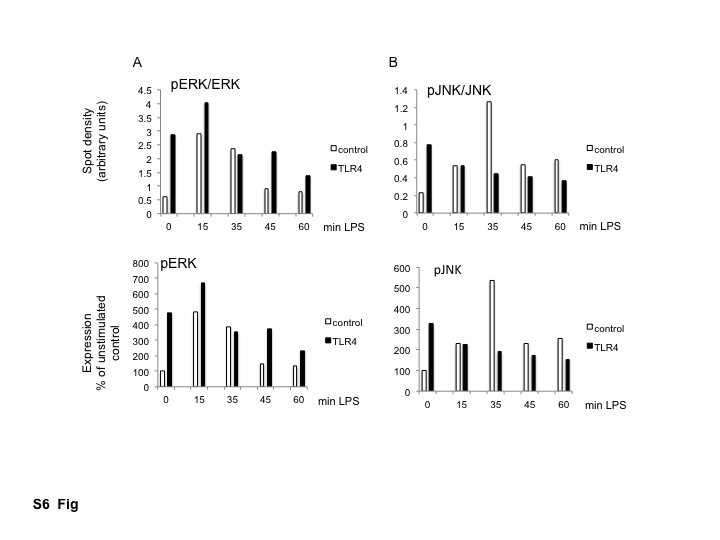

Supplement: S6 Fig — SCC13 TLR4 overexpressing and SCC13pUNO control cells were treated with 10μg/ml ultrapure LPS in a time course of 15min, 35min, 45 min and 60 min. The expression of pERK /ERK and pJNK/JNK was analyzed by western blotting. All quantitative estimations represent the spot density on the entire western blot, which is representative for two independent experiments with reproducible result. (A) Quantitative estimation of pERK before and after LPS treatment. The quantified expression of pERK and ERK is presented as a ratio between the spot density (arbitrary units) of pERK bands and the spot density of the corresponding ERK bands. The differential expression of pERK for every time point is compared to its expression level in untreated cells and presented as “% of control”. (B) Quantitative estimation of pJNK before and after LPS treatment. The quantified expression of pJNK and JNK is presented as a ratio between the spot density (arbitrary units) of pJNK bands and the spot density of the corresponding JNK bands. The differential expression of pJNK for every time point is compared to its expression level in untreated cells and presented as “% of control”. (TIFF) [file pone.0185668.s006.tiff]

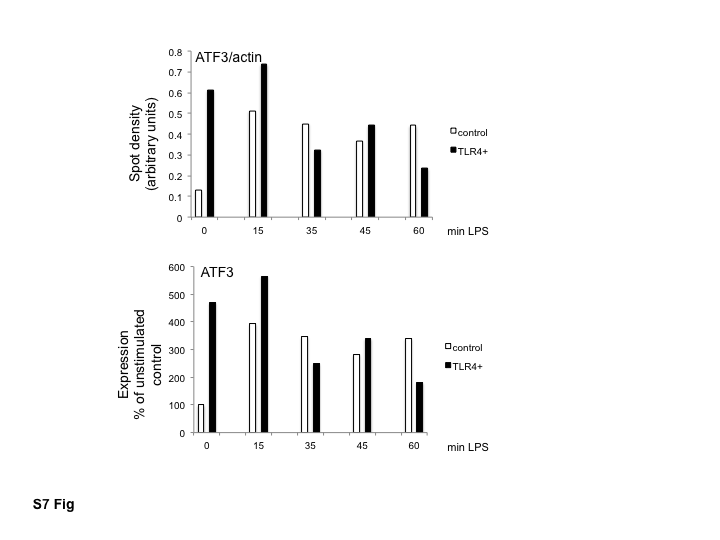

Supplement: S7 Fig — The quantified expression of ATF3 is presented as a ratio between the spot density (arbitrary units) of the ATF3 bands and the spot density of the corresponding actin bands. The differential expression of ATF3 for every time point is compared to its expression level in untreated cells and presented as “% of control”. (TIFF) [file pone.0185668.s007.tiff]

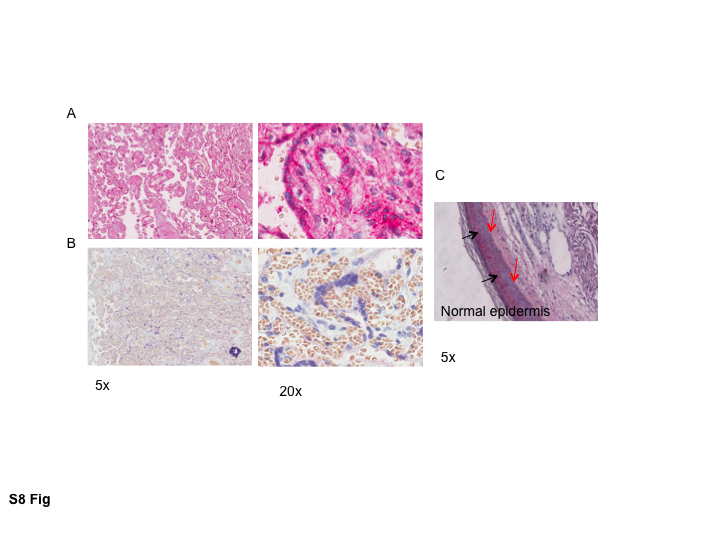

Supplement: S8 Fig — (A) Positive control staining for TLR4 expression in human placenta. The TLR4 detection antibody (HTA 125) was used in a dilution 1:100. The images are presented at magnifications 5X and 20X. The positive red staining represents the TLR4 expression. (B) Negative control staining. The negative control staining was performed on human placenta and in the absence of the TLR4 antibody. (C) Differential TLR4 expression in normal epidermis. The basal keratinocyte layer is characterized by weak red intensity staining for TLR4 (red arrows). Upper and high-differentiated layers show stronger red intensity staining for TLR4 (black arrows). (TIFF) [file pone.0185668.s008.tiff]
